# Supplementary material for: Refined Capsid Structure of Human Adenovirus D26 at 3.4 Å Resolution
Source: Viruses. 2022 Feb 17;14(2):414. doi: 10.3390/v14020414 (PMC8878492; doi:10.3390/v14020414)
Supplement: Supplementary file 1 [file viruses-14-00414-s001.zip › viruses-1537909-supplementary.pdf]

Supplementary Information

Refined capsid structure of human adenovirus D26 at 3.4 Å resolution

Vijay S. Reddy[1*], Xiaodi Yu[1§] and Michael A. Barry[2,3,4,5]
[1]Department of Integrative Structural and Computational Biology, The Scripps Research Institute, La Jolla, CA 92037, USA
[2]Division of Infectious Diseases, [3]Department of Medicine, [4]Department of Immunology, [5]Department of Molecular Medicine, Mayo Clinic, Rochester, MN 55905, USA

[2]Department of Internal Medicine, Division of Infectious Diseases, Mayo Clinic, Rochester, MN 55902, USA

[§]Current address: SMPS, Janssen Research and Development, Spring House, PA 19044, USA

*Corresponding author:
Vijay S. Reddy, E-mail: reddyv@scripps.edu

Table S1. Sequence identity and structural similarity (RMSD) values for VIII pairs in selected HAdVs.

HAdV	Ad26	Ad5 (PDB-ID: 6B1T)	Ad41 (PDB-ID: 6Z7N)
**Ad26**	100 (0)	77.5 (0.61)	81.5 (0.81)
**Ad5**	77.5 (0.61)*	100 (0)	79.3 (0.73)
**Ad41**	81.5 (0.81)	79.3 (0.73)	100 (0)

*Shown in parenthesis are root mean square deviation (RMSD) values for aligned and pruned C-alpha atom pairs estimated using ChimeraX [30].

Table S2. Cryo-EM data and model quality statistics of HAdV-D26 virion.

**Data collection**	
Microscope	FEI Titan Krios
Camera	Gatan K2 Summit
Voltage (kV)	300
Total dose (e-/Å^2)	53
Pixel size	1.31 Å
Defocus range (mm)	0.8-3.0
Micrographs	2,017
Particles selected	37,026
Particles included in final reconstruction	30,834
**Reconstruction**	
Symmetry imposed	I
Resolution (Å) (FSC cut-off: 0.143)	3.38
**Virion model composition and quality statistics**	
Number of chains	31
Number of atoms	105,674
Number of protein residues	13,365
Clash score	15.25
Rotamer outliers	12.26
Molprobity score	3.14
EMRinger Score	2.81
Map-Model CC (main chain)	0.83
Map-Model CC (side chain)	0.81
**Ramachandran statistics**	
Favored	86.62 %
Allowed	13.21 %
Outliers	0.17%
**R.M.S. Deviations**	
Bond lengths (Å)	0.007
Bond angles (°)	0.769

```
CLUSTAL 2.1 multiple sequence alignment

Ad26_Hexon|Q3S8B3       MATPSMMPQWAYMHIAGQDASEYLSPGLVQFARATDTYFSLGNKFRNPTVAPTHDVTTDR
Ad5_Hexon|P04133        MATPSMMPQWSYMHISGQDASEYLSPGLVQFARATETYFSLNNKFRNPTVAPTHDVTTDR
                        **********:****:*******************:*****.******************

Ad26_Hexon|Q3S8B3       SQRLTLRFVPVDREATTYLYKARFTLAVGDNRVLDMASTYFDIRGVLDRGPSFKPYSGTA
Ad5_Hexon|P04133        SQRLTLRFIPVDREDTAYSYKARFTLAVGDNRVLDMASTYFDIRGVLDRGPTFKPYSGTA
                        ********:***** *:* ********************************:********
                                                          HVR1
Ad26_Hexon|Q3S8B3       YNSLAPKGAPNPSQWETKEKQGTTGG------------VQQEKDVTKTFGVAATGGINIT
Ad5_Hexon|P04133        YNALAPKGAPNPCEWDEAATALEINLEEEDDDNEDEVDEQAEQQKTHVFGQAPYSGINIT
                        **:*********.:*:   .    .              * *:: *:.** *. .*****

Ad26_Hexon|Q3S8B3       NQGLLLGTDETAENGKKDIYADKTFQPEPQVGEENWQENEAFYGG-RALKKDTKMKPCYG
Ad5_Hexon|P04133        KEGIQIG-----VEGQTPKYADKTFQPEPQIGESQWYETEINHAAGRVLKKTTPMKPCYG
                        ::*: :*      :*:.  ***********:**.:* *.*  :.. *.*** * ******

Ad26_Hexon|Q3S8B3       SFARPTNEKGGQAKFKPVNEGEQPKDLDIDFAYFDVPGGSPPAGGSGEEYKADIILYTEN
Ad5_Hexon|P04133        SYAKPTNENGGQGILVKQQNGKLESQVEMQFFSTTEATAG-----NGDNLTPKVVLYSED
                        *:*:****:***. :   ::*:  .:::::*     . ..     .*:: ...::**:*:

Ad26_Hexon|Q3S8B3       VNLETPDTHVVYKPGTSDNSSEINLVQQSMPNRPNYIGFRDNFVGLMYYNSTGNMGVLAG
Ad5_Hexon|P04133        VDIETPDTHISYMPTIKEGNSRELMGQQSMPNRPNYIAFRDNFIGLMYYNSTGNMGVLAG
                        *::******: * *  .:..*.  : ***********.*****:****************

Ad26_Hexon|Q3S8B3       QASQLNAVVDLQDRNTELSYQLLLDSLGDRTRYFSMWNSAVDSYDPDVRIIENHGVEDEL
Ad5_Hexon|P04133        QASQLNAVVDLQDRNTELSYQLLLDSIGDRTRYFSMWNQAVDSYDPDVRIIENHGTEDEL
                        **************************:***********.****************.****

Ad26_Hexon|Q3S8B3       PNYCFPLNGTGTNSTYQGVKITNGNDGAEESEWEKDDAISRQNQICKGNVYAMEINLQAN
Ad5_Hexon|P04133        PNYCFPLGGVINTETLTKVKPKTGQ---ENGWEKDATEFSDKNEIRVGNNFAMEINLNAN
                        *******.*. ...*   ** ..*:   *:.  :.   :* :*:*  ** :******:**

Ad26_Hexon|Q3S8B3       LWKSFLYSNVALYLPDSYKYTPANVKLPANTNTYEYMNGRVVAPSLVDAYINIGARWSLD
Ad5_Hexon|P04133        LWRNFLYSNIALYLPDKLKYSPSNVKISDNPNTYDYMNKRVVAPGLVDCYINLGARWSLD
                        **:.*****:******. **:*:***:. *.***:*** *****.***.***:*******

Ad26_Hexon|Q3S8B3       PMDNVNPFNHPRNAGLRYRSMLLGNGRYVPFHIQVPQKFFAIKNLLLLPGSYTYEWNFRK
Ad5_Hexon|P04133        YMDNVNPFNHHRNAGLRYRSMLLGNGRYVPFHIQVPQKFFAIKNLLLLPGSYTYEWNFRK
                         ********* *************************************************

Ad26_Hexon|Q3S8B3       DVNMILQSSLGNDLRVDGASVRFDSVNLYATFFPMAHNTASTLEAMLRNDTHDQSFNDYL
Ad5_Hexon|P04133        DVNMVLQSSLGNDLRVDGASIKFDSICLYATFFPMAHNTASTLEAMLRNDTNDQSFNDYL
                        ****:***************::***: ************************:********

Ad26_Hexon|Q3S8B3       SAANMLYPIPAKATNVPISIPSRNWAAFRGWSFTRLKTKETPSLGSGFDPYFVYSGSIPY
Ad5_Hexon|P04133        SAANMLYPIPANATNVPISIPSRNWAAFRGWAFTRLKTKETPSLGSGYDPYYTYSGSIPY
                        ***********:*******************:***************:***:.*******

Ad26_Hexon|Q3S8B3       LDGTFYLNHTFKKVSIMFDSSVSWPGNDRLLTPNEFEIKRSVDGEGYNVAQCNMTKDWFL
Ad5_Hexon|P04133        LDGTFYLNHTFKKVAITFDSSVSWPGNDRLLTPNEFEIKRSVDGEGYNVAQCNMTKDWFL
                        **************:* *******************************************

Ad26_Hexon|Q3S8B3       VQMLSHYNIGYQGFHVPEGYKDRMYSFFRNFQPMSRQVVDEINYKDYKAVTLPFQHNNSG
Ad5_Hexon|P04133        VQMLANYNIGYQGFYIPESYKDRMYSFFRNFQPMSRQVVDDTKYKDYQQVGILHQHNNSG
                        ****::********::**.*********************: :****: * : .******

Ad26_Hexon|Q3S8B3       FTGYLAPTMRQGQPYPANFPYPLIGQTAVPSVTQKKFLCDRVMWRIPFSSNFMSMGALTD
Ad5_Hexon|P04133        FVGYLAPTMREGQAYPANFPYPLIGKTAVDSITQKKFLCDRTLWRIPFSSNFMSMGALTD
                        *.********:**.***********:*** *:*********.:*****************

Ad26_Hexon|Q3S8B3       LGQNMLYANSAHALDMTFEVDPMDEPTLLYLLFEVFDVVRVHQPHRGVIEAVYLRTPFSA
Ad5_Hexon|P04133        LGQNLLYANSAHALDMTFEVDPMDEPTLLYVLFEVFDVVRVHRPHRGVIETVYLRTPFSA
                        ****:*************************:***********:*******:*********

Ad26_Hexon|Q3S8B3       GNATT
Ad5_Hexon|P04133        GNATT
                        *****
```

Figure S1. Amino acid sequence alignments of hexon subunits from Ad26 and Ad5 viruses

```
CLUSTAL 2.1 multiple sequence alignment


Ad26_PB|A4ZKL2       -MRRAVVSSSPPPSYESVMAQA------------TLEVPFVPPRYMAPTEGRNSIRYSEL
Ad5_PB|P12538        MRRAAMYEEGPPPSYESVVSAAPVAAALGSPFDAPLDPPFVPPRYLRPTGGRNSIRYSEL
                       * *: ...********:: *            .*: *******: ** **********

Ad26_PB|A4ZKL2       APQYDTTRVYLVDNKSADIASLNYQNDHSNFLTTVVQNNDFTPAEASTQTINFDERSRWG
Ad5_PB|P12538        APLFDTTRVYLVDNKSTDVASLNYQNDHSNFLTTVIQNNDYSPGEASTQTINLDDRSHWG
                     ** :************:*:****************:****::*.********:*:**:**

Ad26_PB|A4ZKL2       GDLKTILHTNMPNVNEYMFTSKFKARVMVSRKHPEGVVETDLSQDKLEYEWFEFTLPEGN
Ad5_PB|P12538        GDLKTILHTNMPNVNEFMFTNKFKARVMVSRLPTK------DNQVELKYEWVEFTLPEGN
                     ****************:***.**********  .:       .* :*:***.********

Ad26_PB|A4ZKL2       FSETMTIDLMNNAILENYLQVGRQNGVLESDIGVKFDSRNFKLGWDPVTKLVMPGVYTYE
Ad5_PB|P12538        YSETMTIDLMNNAIVEHYLKVGRQNGVLESDIGVKFDTRNFRLGFDPVTGLVMPGVYTNE
                     :*************:*:**:*****************:***:**:**** ******** *

Ad26_PB|A4ZKL2       AFHPDVVLLPGCGVDFTESRLSNLLGIRKKQPFQEGFRIMYEDLEGGNIPALLDVPKYLE
Ad5_PB|P12538        AFHPDIILLPGCGVDFTHSRLSNLLGIRKRQPFQEGFRITYDDLEGGNIPALLDVDAYQA
                     *****::**********.***********:********* *:*************  *

Ad26_PB|A4ZKL2       SKKKVEDETKNAAAAT------------------------ADTTTRGDTFATPAQET---
Ad5_PB|P12538        SLKDDTEQGGGGAGGSNSSGSGAEENSNAAAAAMQPVEDMNDHAIRGDTFATRAEEKRAE
                     * *.  ::  ..*..:                         * : ******* *:*.

Ad26_PB|A4ZKL2       -----------------AADKKVEVLPIEKDESGRSYNLIQG-THDTLYRSWYLSYTYGD
Ad5_PB|P12538        AEAAAEAAAPAAQPEVEKPQKKPVIKPLTEDSKKRSYNLISNDSTFTQYRSWYLAYNYGD
                                       .:**  : *: :*.. ******.. :  * ******:*.***

Ad26_PB|A4ZKL2       PEKGVQSWTLLTTPDVTCGAEQVYWSLPDLMQDPVTFRSTQQVSNYPVVGAELMPFRAKS
Ad5_PB|P12538        PQTGIRSWTLLCTPDVTCGSEQVYWSLPDMMQDPVTFRSTRQISNFPVVGAELLPVHSKS
                     *:.*::***** *******:*********:**********:*:**:*******:*.::**

Ad26_PB|A4ZKL2       FYNDLAVYSQLIRSYTSLTHVFNRFPDNQILCRPPAPTITTVSENVPALTDHGTLPLRSS
Ad5_PB|P12538        FYNDQAVYSQLIRQFTSLTHVFNRFPENQILARPPAPTITTVSENVPALTDHGTLPLRNS
                     **** ********.:***********:****.**************************.*

Ad26_PB|A4ZKL2       IRGVQRVTVTDARRRTCPYVYKALGIVAPRVLSSRTF
Ad5_PB|P12538        IGGVQRVTITDARRRTCPYVYKALGIVSPRVLSSRTF
                     * ******:******************:*********
```

Figure S2. Amino acid sequence alignments of penton base (PB) subunits from Ad26 and Ad5 viruses

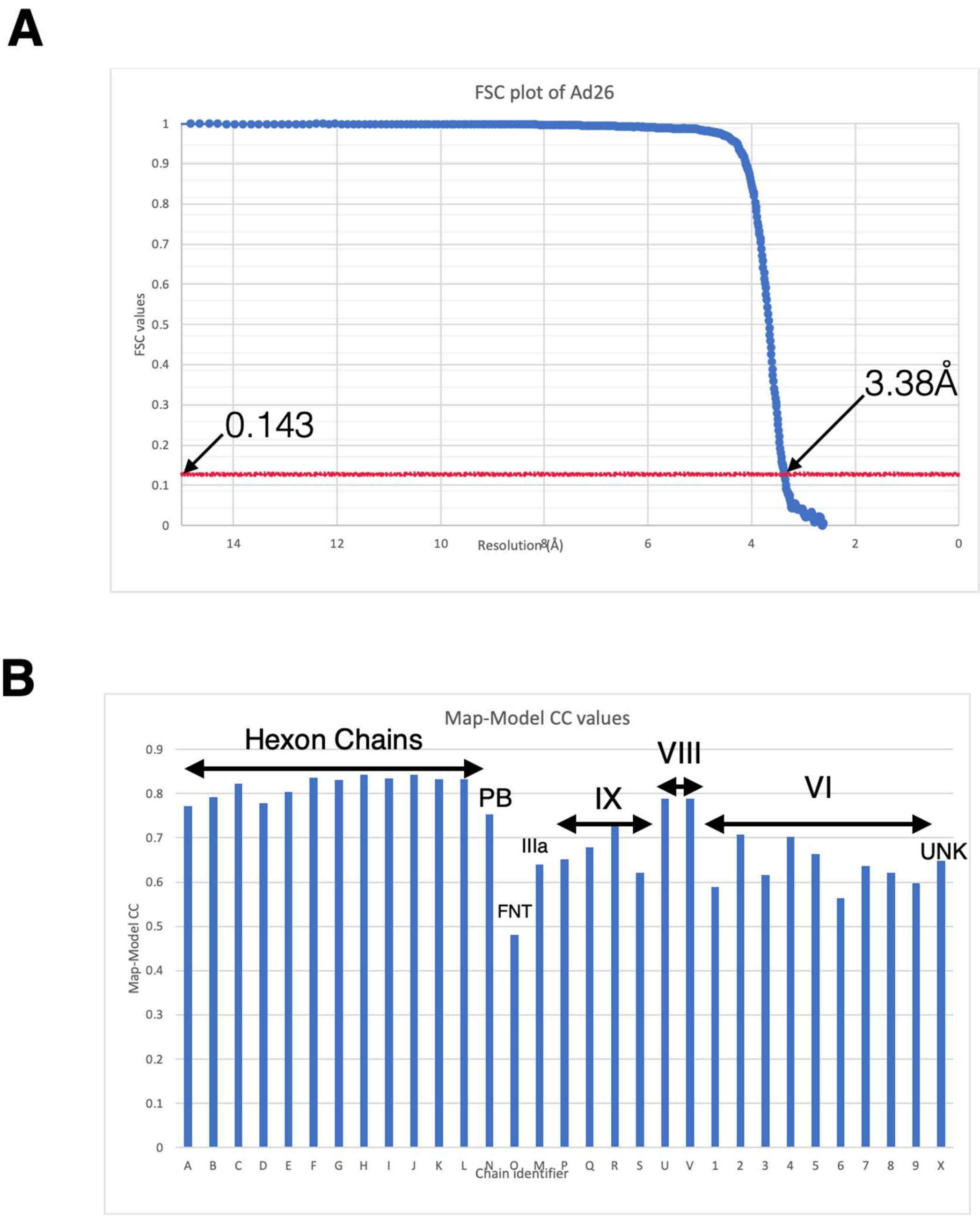

Figure S3. (**A**) FSC plot showing the resolution estimation of the cryo-EM reconstruction of HAdV-D26 (Ad26) virion. The partial FSC values used in the graph as a function of resolution were calculated in cisTEM [60]. (**B**) A bar plot of map-to-model correlation coefficient (CC) values for individual chains reported in the structure of Ad26 virion. The corresponding capsid proteins of the individual chains are identified at the top the bars. The average map-to-model CC values for all the main chain and side chain atoms are 0.83 and 0.81, respectively. The map-to-model CCs were calculated in Phenix [9].
